# Evidence of circulation of Orthobunyaviruses in diverse mosquito species in Kwale County, Kenya

**DOI:** 10.1186/s12985-021-01670-5

**Published:** 2021-10-12

**Authors:** Hellen Koka, Joel Lutomiah, Solomon Langat, Edith Koskei, Albert Nyunja, James Mutisya, Francis Mulwa, Samuel Owaka, Victor Ofula, Samson Konongoi, Fredrick Eyase, Rosemary Sang

**Affiliations:** 1 Centre for Virus Research, Kenya Medical Research Institute, P. O. Box 54628-00200, Nairobi, Kenya; 2 US Army Medical Research Directorate - Kenya, P. O. Box 606-00621, Nairobi, Kenya

**Keywords:** Bunyamwera virus, Nyando virus, Orthobunyaviruses, Mosquitoes

## Abstract

**Background:**

Arbovirus surveillance and recurrence of outbreaks in Kenya continues to reveal the re-emergence of viruses of public health importance. This calls for sustained efforts in early detection and characterization of these agents to avert future potential outbreaks.

**Methods:**

A larval survey was carried out in three different sites in Kwale County, Vanga, Jego and Lunga Lunga. All containers in every accessible household and compound were sampled for immature mosquitoes. In addition, adult mosquitoes were also sampled using CO_2_-baited CDC light traps and BG-Sentinel traps in the three sites and also in Tsuini. The mosquitoes were knocked down using trimethylamine and stored in a liquid nitrogen shipper for transportation to the laboratory where they were identified to species, pooled and homogenized ready for testing.

**Results:**

A total of 366 houses and 1730 containers were inspected. The House Index (HI), Container Index (CI) and Breateau Index (BI) for Vanga Island were (3%: 0.66: 3.66) respectively. In Jego, a rural site, the HI, CI and BI were (2.4%: 0.48: 2.4) respectively. In Lunga Lunga, a site in an urban area, the HI, CI and BI were (22.03%: 3.97: 29.7) respectively. The indices suggest that this region is at risk of arbovirus transmission given they were above the WHO threshold (CI > 1, HI > 1% and BI > 5). The most productive containers were the concrete tanks (44.4%), plastic tank (22.2%), claypot (13.3%), plastic drums (8.9%), plastic basins (4%), jerricans (1.2%) and buckets (0.3%). Over 20,200 adult mosquitoes were collected using CDC light traps, and over 9,200 using BG- sentinel traps. These mosquitoes were screened for viruses by inoculating in Vero cells. Eleven Orthobunyavirus isolates were obtained from pools of *Ae. pembaensis* (4), *Ae. tricholabis* (1), *Cx. quinquefasciatus* (3), *Culex* spp. (1) and *Cx. zombaensis* (2). Five of the Orthobunyaviruses were sequenced and four of these were determined to be Bunyamwera viruses while one isolate was found to be Nyando virus. One isolate remained unidentified.

**Conclusions:**

These results indicate circulation of Orthobunyaviruses known to cause diverse grades of febrile illness with rash in humans in this region and highlights the need for continued monitoring and surveillance to avert outbreaks.

## Introduction

Arboviruses are a group of diverse RNA viruses transmitted by arthropods in class *Insecta* and *Arachnida* that include mosquitoes, sandflies, biting midges and ticks. There are over 500 species of arboviruses worldwide that cause disease in vertebrates and these have been classified into six families, namely: *Peribunyaviridae, Flaviviridae, Togaviridae, Reoviridae, Rhabdoviridae* and *Orthomyxoviridae*. The *Peribunyaviridae* family of arboviruses comprise 5 genera: *Orthobunyavirus, Phlebovirus, Nairovirus, Hantavirus* and *Tospoviru*s [2]*.* The genomic structure of Orthobunyaviruses consists of a single stranded negative sense RNA, that is tri-segmented; with S (small), M (medium) and L (large) segments [13]. Apart from the *Tospovirus* genus which mainly comprises of plant viruses, viruses in the other genera in this family infect mammals and cause fever, encephalitis, haemorrhagic fever and an acute respiratory illness [8]. The Orthobunyavirus genus includes 18 serogroups. Among these is the Bunyamwera serogroup which is the largest and consists of the Bunyamwera virus (BUNV), Ngari virus (NRIV) and Batai virus (BATV), among others [41].

The BUNV was originally isolated from *Aedes spp.* mosquitoes collected in the Semiliki Forest in Uganda in 1943 [35] and later in 1955 from *Ae. circumluteolus* mosquitoes and man in South Africa [18, 19] and is the most commonly isolated orthobunyavirus in Africa. On the other hand, NRIV was isolated in 1979 from *Ae. simpsoni* mosquitoes in Senegal and from humans in 1993 [42]. It was also reported in Sudan in 1988 and in Northeastern Kenya in 1997/98, Tanzania and Somalia in 1997–1998 and is a natural reassortant of BUNV and BATV with the S and L segments from the Bunyamwera virus and the M segment from Batai virus [3, 4, 9, 40]. NRIV is very virulent and has been associated with hemorrhagic fever so far the molecular basis of the virulence has not been established [11]. Studies in Kenya have indicated that BUNV and NRIV are circulating in mosquitoes and ticks collected in Garissa, Isiolo, Magadi and Tana delta [27]. BUNV has also been isolated from ticks collected in northeastern Kenya [24] and recently from mosquitoes collected in Lake Victoria Basin [1]. In contrast, BATV was first isolated from *Culex* mosquitoes in Malaysia in 1955 [43]. Since then, the virus has been detected in mosquitoes in Germany [33] and Italy [16]. It has also been isolated from human and bovine blood in Sudan and Japan respectively [25, 40]. This virus causes a mild febrile flu-like illness in man and animals [17]. This virus has not been isolated in Kenya despite results from a sero-survey in three health facilities showing cross neutralization in serum samples that suggested patients who were seropositive for NRIV may have been infected with BATV [28]. Notably, Nyando virus (NDV) is an Orthobunyavirus that is distinct from those in the Bunyamwera serogroup. As such, it has been classified on its own in the Nyando serogroup [39]. NDV is associated with febrile illness, myalgia and vomiting, but only one case of human infection in Central Africa has been reported thus far. Additionally, it has been isolated from *An. funestus* in Kenya and Uganda, from *Eretmapodites* spp. in Ethiopia and from *Aedes* spp. in Cameroon [12].

Outbreaks of arboviruses are increasingly being reported in Kenya, particularly in the Coastal region where chikungunya fever and dengue fever outbreaks have become frequent [20, 23]. Most of these outbreaks in the Coastal region have been reported in Mombasa County. For this, study, the arbovirus surveillance was carried out in Lunga Lunga sub-county, Kwale County in the Coastal region. The proximity of Kwale County to Mombasa County and coast of Tanzania where outbreaks of dengue have also occurred premised this region as likely to be experiencing undetected circulations of arboviruses like dengue, chikungunya and others. Hence the survey was conducted to establish the extent of the public health menace across the coastline of Kenya.

## Methods

### Study site description

**Fig. 1:**
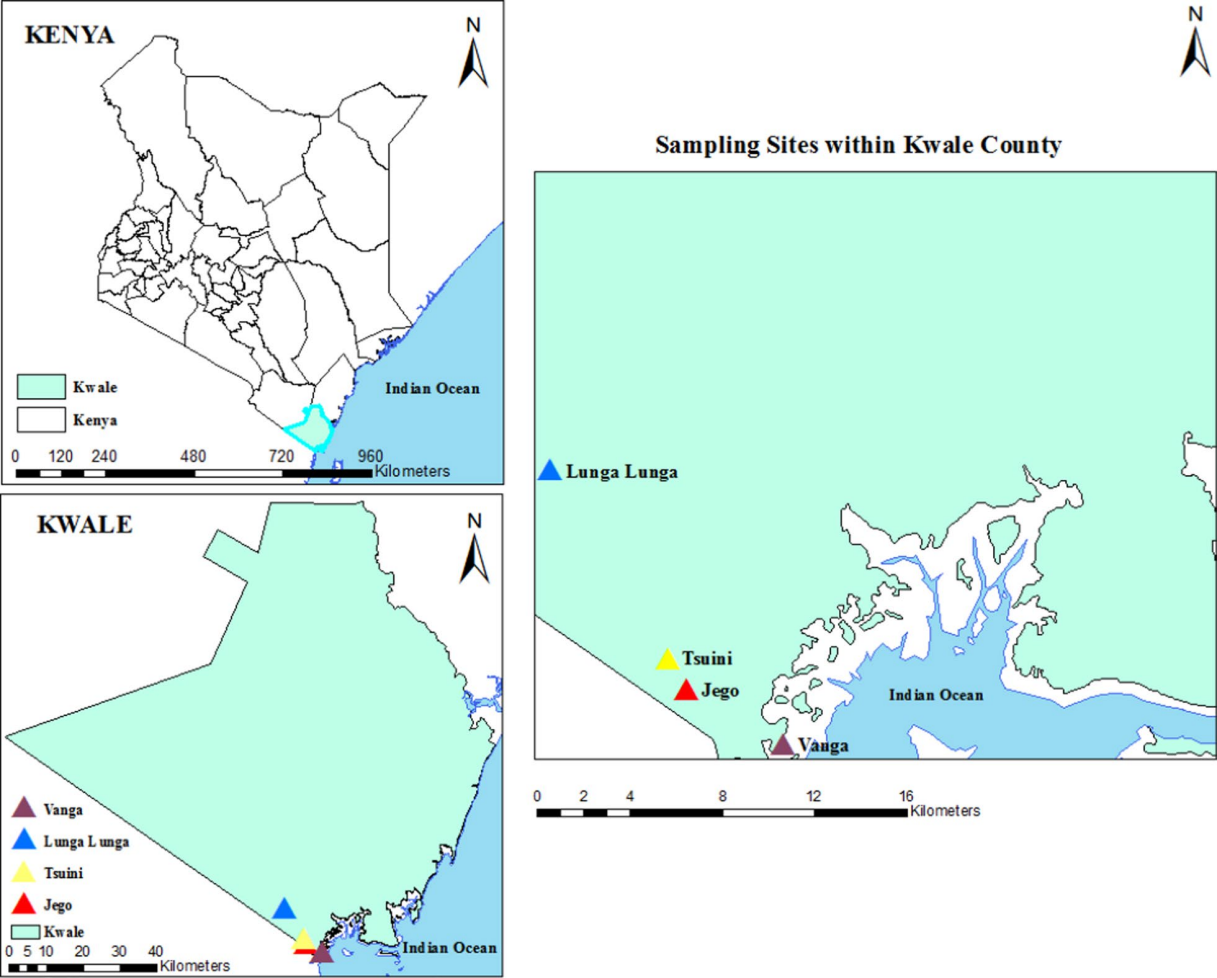
Map of Kwale County with showing the sites in this study

This study was carried out in Lunga Lunga sub-county, Kwale County (Fig. 1). Kwale County covers an area of 8270.2 km^2^ and comprises of four sub-counties: Matuga, Msabweni, Kinango and Lunga Lunga. Lunga Lunga sub-county is located six kilometres from the Kenya border with Tanzania. The population of Lunga Lunga sub-county based on the 2019 census is approximately 198,423 persons. Vanga Island is a coastal fishing settlement, while Jego and Tsuini represent a rural village. On the other hand, Lunga Lunga is an urban area. The economic activities in this sub-county include, fishing, farming, sand harvesting and small-scale trading [34].

### Mosquito sampling

Mosquitoes were sampled in Vanga Island from 27th July–1st Aug 2017. A second sampling was done in Vanga Island from the 15th–17th Sep 2018, in Jego which is a rural setting on the Vanga mainland from 14th–19th Sep 2018 and in Tsuini from 18th Sep 2018. A third sampling was conducted in Lunga Lunga, from the 13th–20th Dec 2019. Lunga Lunga is an urban commercial centre located on the mainland and serving as the commercial center for the sub-county as well as the border crossing into Tanzania. The last site to be sampled was Tsuini on 20th Dec 2019. Adult mosquitoes were collected in sites randomly selected within the locations, while larvae were collected in Vanga Island and Jego from the 14th–19th Sept 2018, a dry season while in Lunga Lunga, the survey was done from 13th–20th Dec 2019, a wet period.

### Larval survey of *Aedes aegypti*

In households that were accessible, water-holding containers found indoors and outdoors were inspected for mosquito immatures. Samples were collected from each productive container using ladles or Pasture pipettes. A score was given to every container inspected as either wet negative or wet positive depending on presence of and number of *Ae. aegypti* immatures found [37]. The immature mosquitoes collected were reared to adulthood and identified to species [7] so that only *Ae. aegypti* mosquitoes were used to calculate larval indices. The following larval indices were calculated to determine the risk of arbovirus transmission,*House index (HI)*: percentage of houses where breeding larvae and/or pupae where found. *Container index (CI)*: percentage of water-holding containers infested with larvae or pupae. *Breteau index (BI)*: number of positive containers per 100 houses inspected [29].

### Adult mosquito collections

Adult mosquitoes were collected using CO_2_-baited CDC light traps (John W Hock) and BG-Sentinel traps (Biogents). The CDC light traps were hung randomly on selected sites near houses or at the edges of the compound, 10 traps per night. Each trap was baited with 0.5 kg of CO_2,_ hung from dusk to dawn and retrieved in the morning. The BG- Sentinel traps were set from morning to evening outside houses and retrieved in the evening [23]. At a temporary laboratory set up in the field station, the mosquitoes collected from each of the traps were knocked down using triethylamine. The mosquitoes were sorted and identified morphologically using mosquito identification keys and pooled (≤ 25 mosquitoes per pool) by species, sex and collection site [10, 14]. Each mosquito pool was stored in a 1.5-ml cryogenic vial. The cryogenic vials were stored in liquid nitrogen and transferred to the level-2 biosafety laboratory based at the Kenya Medical Research Institute’s (KEMRI) centre for virus isolation.

### Mosquito processing and virus isolation

The mosquito pools obtained were homogenized using Minimum Essential Medium supplemented with Foetal Bovine Serum (Gibco), L-Glutamine and antibiotics (10,000 units penicillin, 10 mg streptomycin and 25 μg amphotericin B per ml-Sigma) [23]. Homogenates were clarified by centrifugation at 12000 rpm for 10 min and the resultant supernatants inoculated in 24 -well plates of Vero cells (CCL-81™) in supplemented Minimum Essential Medium [27]. The cultures were incubated at 37 °C and monitored for cytopathic effect (CPE) daily for 14 days. Cultures showing CPE were harvested and viruses identified by RT-PCR and sequencing.

### RNA extraction and reverse transcription

RNA was extracted from cell culture isolates using Trizol^®^LS Chloroform method. The RNA extract was transcribed into cDNA using First Strand cDNA synthesis kit (Invitrogen) with random hexamers, followed by Polymerase Chain Reaction (PCR) using Amplitaq Gold PCR mastermix (Applied Biosystems). Amplification by PCR used universal arbovirus primers targeting the genus of Flavivirus, Orthobunyavirus and Alphaviruses [27].

### Characterization by sequencing

Preliminary characterization of the isolates was performed by Sanger sequencing. Amplicons were cleaned using DNA Clean & Concentrator Kit (ZymoResearch, US). Chromatogram files were prepared and edited to generate consensus sequences using BioEdit v7.2.5 [15].

A subset of the samples was also prepared for whole genome sequencing. RNA extracts were used as input to perform library preparation by using Truseq mRNA Library Prep kit (Illumina, San Diego, CA, USA), following the manufacturer’s recommended protocol which was modified to exclude the mRNA clean-up steps [5]. The prepared libraries were sequenced on an Illumina Miseq platform (Illumina, San Diego, CA, USA) using a 2 × 300 base paired-end reads. Sequence analysis was performed by making use of NGS Mapper v1.5 pipeline (ngs_mapper). The pipeline performs a number of sequential steps on the raw sequence reads, which includes; adapter trimming, raw sequence read cleanup, reference-based mapping and assembly and finally consensus sequence generation. The sequences reported in this study are available in GenBank under accession numbers MW314021-MW314035.

### Sequence analysis and phylogenetics

Sequences generated in this study were analyzed using NCBI Blast and phylogenetic analysis was performed using MEGA7 [21]. Sequences falling under the different Orthobunyavirus serogroups were downloaded from Genbank and combined with those generated in this study. Alignment of the individual segments was achieved with Muscle v6 embedded in MEGA7, and phylogenetic analysis was performed on each of the segments using the Maximum Likelihood approach in MEGA7. Reassortment was evaluated by examining the topology of the phylogenetic trees generated for each of the individual segments.

### Statistical analysis

All analyses were performed using R version 3.6.1 [30]. For larval data, we summarized the larval indices separately for each site. For adult mosquito data, we summarized the collections by collection method (i.e., CDC-light traps and BG sentinel traps) and by site. We also produced mosquito richness and relative abundance of species separately for each site and combined. Data on proportions (or percentages) were compared across the sites using Chi Square test. Multiple comparison of proportions was adjusted for using Holm’s method. All tests were performed at 5% significance level.

## Results

### Container type and positivity for *Ae. aegypti* larvae

In Vanga Island, 164 houses were sampled, of which 5 had positive water containers for *Ae. aegypti* larvae/pupae with a HI of 3%. Six out of a total of 903 containers inspected indoors and outdoors were positive, with a CI of (0.66) and BI of (3.66). In Jego village, 2 out of 84 inspected houses were had positive water containers, with a HI of 2.4%. A total of 414 containers were inspected indoors and outdoors and 2 were positive, with a CI of (0.48) and a BI of (2.4). In Lunga Lunga an urban area, 118 houses were sampled of which 26 were positive giving a HI of 22.03%. A total of 413 containers were inspected indoors and outdoors and 35 were positive, with a CI of (8.5) and a BI of (29.7) (Table 1).

**Table 1:** Larval indices for *Ae. aegypti* for the three sites

Site	Index*		
	House Index	Container Index	Breateau Index
Vanga Island	3.05% (5/164)	0.66% (6/903)	3.66
Jengo Village	2.40% (2/84)	0.48% (2/414)	0.02
Lunga Lunga	22.03% (26/118)	8.50% (35/413)	0.30

^*^House index: percentage of houses where breeding larvae and/or pupae where found; Container index: percentage of water-holding containers infested with larvae or pupae; Breteau index: number of positive containers per 100 houses inspected

**Fig. 2:**
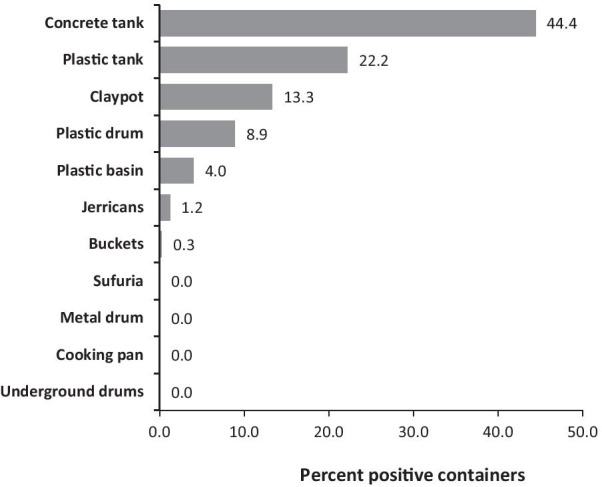
Immature *Ae. aegypti* positivity from diverse containers collected in the three sites

In the three sites, a total of 1,730 containers were inspected indoors and outdoors (Table 2). The most abundant containers were the buckets 42.7%, jerricans 32.9%, plastic drums 14.2% and plastic basins 5.8%. The most productive containers – measured in terms of proportions of inspected containers which are positive – were the concrete tanks (44.4%), plastic tank (22.2%), claypot (13.3%), plastic drums (8.9%), plastic basins (4%), jerricans (1.2%) and buckets (0.3%) (Fig. 2).

**Table 2:** Immature *Ae.aegypti* positivity from diverse containers collected indoors and outdoors in the three sites

Container type	Vanga		Jego		LungaLunga		Combined	
	N	n	N	n	N	n	N	n
Jerricans	154	0	207	0	209	7	570	7
Buckets	559	0	172	0	7	2	738	2
Plastic drum	84	0	32	2	131	20	247	22
Underground drums	4	0	0	0	0	0	4	0
Plastic tank	0	0	1	0	8	2	9	2
Plastic basin	57	2	2	0	41	2	100	4
Cooking pot	10	0	0	0	0	0	10	2
Metal drum	4	0	0	0	0	0	4	0
Concrete tank	21	4	0	0	0	0	21	4
Claypot	0	0	0	0	15	2	15	2
Tire	2	0	0	0	2	0	4	0
Borehole	8	0	0	0	0	0	8	0
Total	903	6	414	2	413	35	1730	43

N = number of containers inspected; n = number of positive water containers

### Adult mosquito

A total of 29,447 mosquitoes, belonging to 7 genera and 36 species, were collected by CDC light traps (20,236) and by BG sentinel traps (9,211). After identification and pooling, 1,950 pools were obtained. The greatest diversity was in the genus *Aedes* that recorded 13 species, followed by *Culex* (9), *Anopheles* (7), *Coquilletidia* (3), *Mansonia* (2), *Eretmapodite* (1) and *Filcabia* (1). The species richness was 33 in Lunga Lunga, 30 in Tsuini, 28 in Vanga and 28 in Jego. Seventeen species were recorded in all four sites, while 9 were recorded in single sites only. More *Aedes, Culex* and *Mansonia* were collected in Vanga Island than other sites while more *Anopheles* species were collected in Tsuini. Mosquito abundance varied significantly across the sites (Chi Sq. = 892.8, *df* = 3, *p* < 0.001): Vanga recorded significantly larger numbers (50.2%) than all other three sites (*p* < 0.001). Tsuini recorded higher abundance (19.2%) than Lunga Lunga (16%; p = 0.018) and Jego (14.6%; *p* < 0.001). There was no significant difference in abundance between Lunga Lunga and Jego (*p* = 0.229). Table 3 exhibits the relative abundance of these mosquitoes separately for each site and all sites combined. The table shows that *Cx. quinquefasciatus * was the overall most abundant species sampled in all the sites combined (17.8%), followed by *Ae. pembaensis* (11.9%), *An. funestus* (11.2%), *Cx. annuloris* (9%), *Mn. uniformis* (8.9%) and *Ae. tricholabis* (6.3%). The least overall sampled species were *Ae. africana*, *Ae. vittatus*, *Coq. fuscopennata*, *Coq. metallicus* and *Cx. tigripes*.

**Table 3:** Relative abundance of adult mosquito species for four site in Kwale County

Species	Site									
	Jego		Lunga Lunga		Tsuini		Vanga		Combined	
	Nr	%	Nr	%	Nr	%	Nr	%	Nr	c%
*Cx. quinquefasciatus*	24	8.5	40	12.8	30	8.0	254	25.9	348	17.8
*Ae. pembaensis*	35	12.3	2	0.6	26	6.9	170	17.4	233	11.9
*An. funestus*	30	10.6	13	4.2	123	32.8	53	5.4	219	11.2
*Cx. annulioris*	30	10.6	6	1.9	50	13.3	90	9.2	176	9.0
*Mn. uniformis*	54	19.0	8	2.6	6	1.6	106	10.8	174	8.9
*Ae. tricholabis*	33	11.6	7	2.2	22	5.9	61	6.2	123	6.3
*Mn. africana*	29	10.2	13	4.2	11	2.9	33	3.4	86	4.4
*Cx. zombaensis*	1	0.4	4	1.3	1	0.3	71	7.3	77	3.9
*Ae. furfurea*	3	1.1	53	17.0	5	1.3	0	0.0	61	3.1
*An. coustani*	17	6.0	6	1.9	7	1.9	24	2.5	54	2.8
*Ae. ochraceus*	0	0.0	32	10.3	14	3.7	0	0.0	46	2.4
*Ae. aegypti*	1	0.4	16	5.1	2	0.5	26	2.7	45	2.3
*An. gambiae*	0	0.0	8	2.6	11	2.9	14	1.4	33	1.7
*An. squamosus*	0	0.0	21	6.7	9	2.4	2	0.2	32	1.6
*Cx. vansomereni*	8	2.8	8	2.6	3	0.8	9	0.9	28	1.4
*Ae. mcintoshi*	0	0.0	11	3.5	13	3.5	3	0.3	27	1.4
*Ae. sudanensis*	0	0.0	14	4.5	13	3.5	0	0.0	27	1.4
*Cx. univittatus*	4	1.4	7	2.2	2	0.5	11	1.1	24	1.2
*Ae. tarsalis*	1	0.4	9	2.9	8	2.1	4	0.4	22	1.1
*Culex*	3	1.1	2	0.6	2	0.5	14	1.4	21	1.1
*Aedes*	1	0.4	3	1.0	1	0.3	14	1.4	19	1.0
*Ae. hirsutus*	2	0.7	4	1.3	2	0.5	3	0.3	11	0.6
*Cx. poicilipes*	0	0.0	2	0.6	7	1.9	2	0.2	11	0.6
*Ae. simpsoni*	2	0.7	3	1.0	1	0.3	3	0.3	9	0.5
*Eret. chrysogaster*	0	0.0	2	0.6	1	0.3	4	0.4	7	0.4
*Cx. ethiopicus*	0	0.0	5	1.6	1	0.3	0	0.0	6	0.3
*Anopheles*	0	0.0	3	1.0	1	0.3	1	0.1	5	0.3
*Fi. medioneata*	3	1.1	0	0.0	0	0.0	1	0.1	4	0.2
*Ae. metallicus*	0	0.0	2	0.6	1	0.3	0	0.0	3	0.2
*An. nili*	0	0.0	3	1.0	0	0.0	0	0.0	3	0.2
*An. pharoensis*	0	0.0	1	0.3	0	0.0	2	0.2	3	0.2
*An. maculipalpis*	0	0.0	1	0.3	0	0.0	1	0.1	2	0.1
*Coq. aurites*	0	0.0	0	0.0	0	0.0	2	0.2	2	0.1
*Cx. cinereus*	0	0.0	2	0.6	0	0.0	0	0.0	2	0.1
*Filcabia*	2	0.7	0	0.0	0	0.0	0	0.0	2	0.1
*Ae. africana*	0	0.0	0	0.0	1	0.3	0	0.0	1	0.1
*Ae. vittatus*	0	0.0	1	0.3	0	0.0	0	0.0	1	0.1
*Coq. fuscopennatus*	0	0.0	0	0.0	0	0.0	1	0.1	1	0.1
*Coq. metallicus*	1	0.4	0	0.0	0	0.0	0	0.0	1	0.1
*Cx. tigripes*	0	0.0	0	0.0	1	0.3	0	0.0	1	0.1
Grand Total	284		312		375		979		1950	

^*^Nr is number of mosquitoes

### Virus isolation and sequencing data

**Fig. 3:**
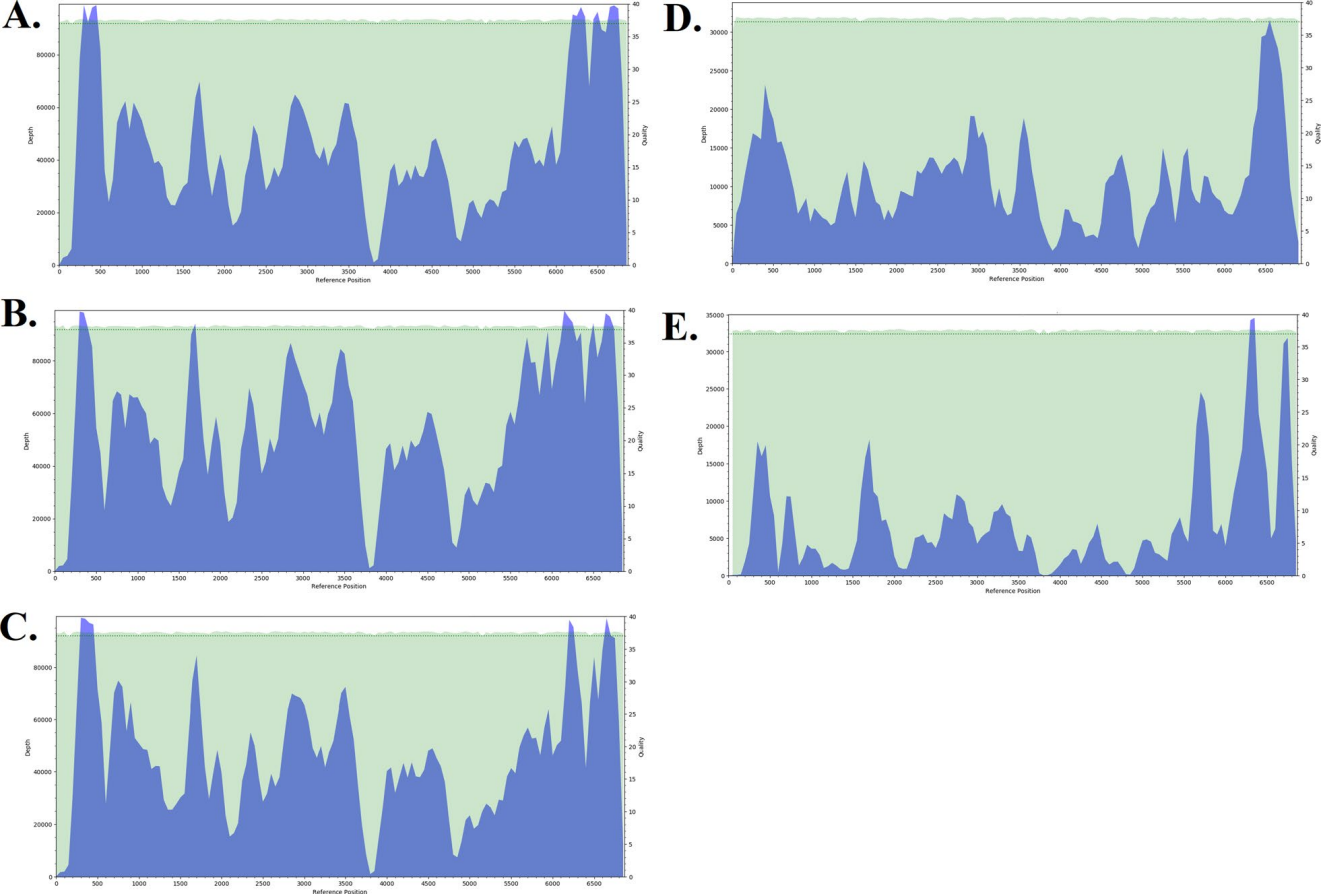
Plots showing the depth of coverage for the L segment of **A** KW_S1_24873, **B** KW_S1_25399, **C** KW_S1_25204, **D** KW_S1_25175 and **E** KW_S1_25397. Similar coverage was observed across the other segments of the respective isolates

**Fig. 4:**
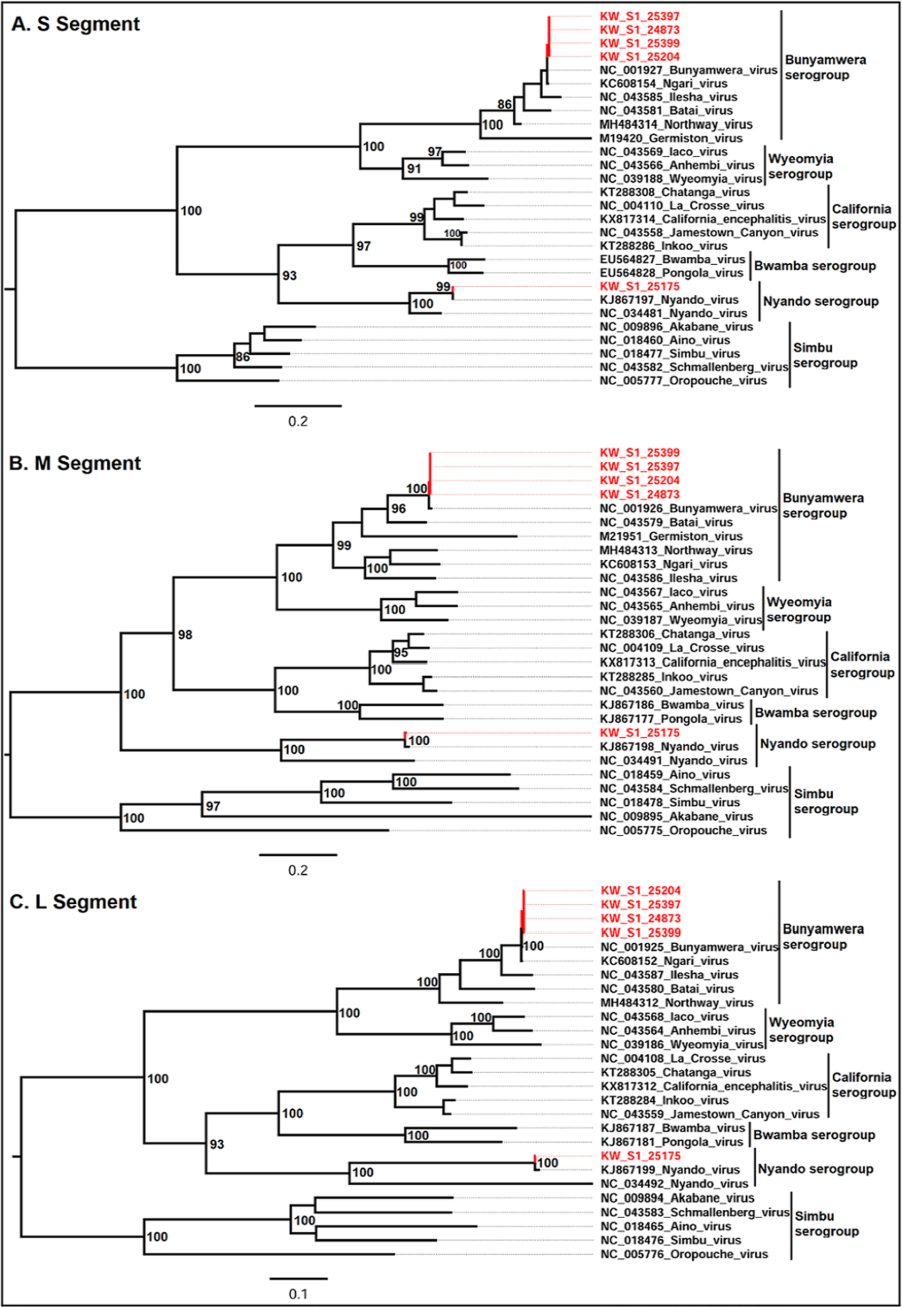
Mid-point rooted maximum likelihood phylogeny for **A** S Segment, **B** M Segment and **C** L segment. The trees were generated based on sequences belonging to 5 different serogroups of bunyaviruses. Sequences obtained in this study are colored with red tip-labels in the tree

There was no virus isolated from immature mosquitoes. However, 12 isolates were obtained from the 1950 pools of adult mosquitoes that were tested. Ten of the pools tested positive for Bunyavirus using the California-Bunyamwera serogroup primers [22] while the two other isolates remained unidentified by Flavivirus, Bunyavirus and Alphavirus universal primers. Sanger sequencing of the successfully amplified isolates resulted in generation of sequences that showed approximately 99% sequence homology to Bunyamwera and Ngari viruses. Considering the primers we used only targets a region within the small segment (S segment) of the Bunyamwera serogroup, it was unclear whether the identity of the virus was a Bunyamwera or Ngari virus. Therefore, we performed whole genome sequencing on four of the Bunyavirus isolates including those obtained from *Ae. pembaensis* (2), *Cx. quinquefasciatus* (1), and *Culex spp* (1) as well as one isolate from the unidentified group that was obtained from *Cx. zombaensis*. Full genome sequences were obtained from all the 5 isolates, including all the three segments of the individual isolates. The depth of coverage across the entire segments of all the 5 genomes was well covered, with the lowest being 24X (Fig. 3). All the other coding regions of the genomes had a high coverage (Fig. 3). All the three segments of the 4 Bunyamwera isolates showed a high similarity to the Bunyamwera virus (NC_001925, NC_001926, NC_001927) with amino acid (aa) similarity of approximately 98.6% (M segment), 99.7% (L segment) and 99.5% (S segment). This, therefore, confirms the identity of this virus as a Bunyamwera virus. The virus from the unknown isolate showed a high similarity to Nyando virus (KJ867197.1, KJ867198.1, KJ867199.1) with percent (aa) similarity of 100% (S segment), 98.05% (M segment), and 98.94% (L segment). The sequences generated in this study were aligned and compared with 23 other sequences belonging to the different serogroups including Bunyamwera, Wyeomyia, California, Bwamba, Nyando and Simbu serogroups which were obtained from Genbank. Maximum likelihood phylogenetic analysis using the individual segments of this set of sequences placed the 4 isolates into the Bunyamwera serogroup and one isolate into the Nyando serogroup, particularly with close similarity to the Bunyamwera virus and Nyando virus respectively. There was no incongruities observed on the different phylogenies generated based on the individual segments (Fig. 4).

## Discussion

Entomological surveillance in Kwale County was preceded by the dengue and chikungunya outbreaks that occurred in Mombasa County between 2017 and 2018. With this in mind, the aim of the study was to evaluate entomological risk factors for transmission of these viruses in Kwale County and assess the risk for outbreaks. The findings of this study, have revealed that Vanga Island and Jego were at medium risk for dengue and chikungunya virus transmission based on the HI observed. However, in Lunga Lunga, all the indices exceeded the WHO thresholds for the risk of dengue suggesting that this urban area was at greater risk and has a potential for transmission of any virus that is transmitted by *Ae. aegypti* mosquitoes [31]. The higher risk level at the Lunga Lunga site could have been as a result of sampling the immatures in the wet season as compared to Vanga Island and Jego that were sampled in the dry season. The three sites, had diverse water holding containers both indoors and outdoors with clean water that provided a perfect breeding habitat for *Ae. aegypti*, thus, increasing the risk of dengue and chikungunya among other arboviruses [36, 38].

It should be noted that all the three sites had diverse mosquito species. However, Vanga Island, had a greater diversity of mosquito species which could be attributed to the fact that mosquito sampling was done twice in this site. Although Tsuini was sampled twice, the mosquito density was lower and similar to the sites that were only sampled once. In as much as, Lunga Lunga was sampled during the wet season, the mosquito density was lower compared to the sites that were sampled in the dry season. It thus appears that absence of rain, may not affect mosquito abundance due to availability of diverse water storage containers, such as the concrete tanks in most homes, which provide conducive breeding habit. *Ae. pembaensis, Cx.*. *quinquefasciatus, Cx. annuloris, Mn. uniformis* and *Ae. tricholabis* were the most abundant species in all the sites. These species have all been implicated in previous reports for arbovirus transmission in different parts of the country and this strongly suggests that the sites in this study are at risk [27, 32].

Although dengue and chikungunya viruses were not detected in the three sites during the sampling period, two viruses, BUNV and NDV, in the genus Orthobunyavirus were isolated from mosquitoes collected in Vanga Island. BUNV has been isolated before from *An. funestus*, *Ae. mcintoshi* and *Ae. tricholabis* mosquitoes collected in Magadi and Garissa respectively [27] whereas NDV has previously been isolated from *An. funestus*, *Eretmapodite*s spp. and *Aedes* spp. [12]. Undoubtedly, the isolation of BUNV from *Ae. pembaensis*, *Cx. quinquefasciatus and Culex spp.* and NDV from *Cx. zombaensis w*idens the potential vector species bracket involved in the transmission of these viruses. An overview of the Sanger sequencing data shows that, nine of the BUNV isolates were identified from female mosquitoes while one came from a pool of male *Ae. pembaensis* mosquitoes, providing evidence of possible vertical transmission of the virus in this species. A limitation of this study is that whole genome sequencing could not be done on all the isolates. In reference to the whole genome sequences on five of the isolates from this study, the topology of the trees for the three segments was similar suggesting no segment reassortment had occurred among these isolates [6].

Overall, the larval indices and virus isolations in this study, greatly suggest that the Lunga Lunga sub-county is at high risk of diverse arbovirus transmission. Public health efforts are concentrated in Mombasa and Kilifi where there is greater risk and repeated reports of outbreaks of dengue and chikungunya. These findings are important because reassortments and recombinations occur more frequently in Orthobunyaviruses and may potentially cause the emergence of new viruses of greater public health importance in Kwale County.

## Conclusions

Briefly, the larval indices indicate that Lunga Lunga sub-county is at risk of dengue transmission. The residents of Lunga Lunga sub-county have an unreliable supply of water that necessitates use of water holding containers as documented by World Bank Environmental and Social Impact Assessment Report of July 2019 [39]. The water holding containers, in turn, have provided perfect breeding ground for mosquito larvae. We recommend the sensitization of the communities on handling water storage containers to avoid mosquito breeding. The government should also work on providing a reliable water supply. This study also determined that BUNV and NDV, arboviruses known to cause febrile illness, were circulating in this region. It also highlighted the potential risk for emergence of other arboviral diseases since a variety of susceptible vectors are present. There was also evidence that BUNV may be maintained transovarially with potential for the virus to be continuously maintained in the environment. Further, the isolation of Orthobunyaviruses highlights the need for further studies to be carried out in this area to understand virus-vector -host transmission dynamics. Comprehensive assessment of arbovirus risk should also be further assessed through a sero-survey to establish relative risk to public health.

## Data Availability

All data generated or analyzed in this study are included in this published article and its supplementary files.

## Abbreviations

BUNV: Bunyamwera virus
NDV: Nyando virus
BATV: Batai virus
NRIV: Ngari virus
RNA: Ribonucleic acid
PCR: Polymerase chain reaction
WHO: World Health Organization
HI: House Index
CI: Container Index
BI: Breateau

## References

[1] Ajamma YU, Onchuru TO, Ouso DO, Omondi D, Masiga DK, Villinger J (2018). Vertical transmission of naturally occurring Bunyamwera and insect-specific flavivirus infections in mosquitoes from islands and mainland shores of Lakes Victoria and Baringo in Kenya. PLoS Neglect Trop Diseases.

[2] Blitvich BJ, Beaty BJ, Blair CD, Brault AC, Dobler G, Drebot MA, Haddow AD, Kramer LD, LaBeaud AD, Monath TP, Mossel EC, Plante K, Powers AM, Tesh RB, Turell MJ, Vasilakis N, Weaver SC (2018). Bunyavirus Taxonomy: Limitations and Misconceptions Associated with the Current ICTV Criteria Used for Species Demarcation. Am J Trop Med Hyg.

[3] Bowen MD, Trappier SG, Sanchez AJ, Meyer RF, Goldsmith CS, Zaki SR, Dunster LM, Peters CJ, Ksiazek TG, Nichol ST (2001). A reassortant bunyavirus isolated from acute hemorrhagic fever cases in Kenya and Somalia. Virology.

[4] Briese T, Bird B, Kapoor V, Nichol ST, Lipkin WI (2006). Batai and Ngari viruses: M segment reassortment and association with severe febrile disease outbreaks in East Africa. J Virol.

[5] Clark JJ, Gilray J, Orton RJ, Baird M, Wilkie G, Filipe AS, Johnson N, McInnes CJ, Kohl A, Biek R (2020). Population genomics of louping ill virus provide new insights into the evolution of tick-borne flaviviruses. PLoS Negl Trop Dis.

[6] Dutuze MF, Nzayirambaho M, Mores CN, Christofferson RC. A Review of Bunyamwera, Batai, and Ngari Viruses: Understudied Orthobunyaviruses With Potential One Health Implications. Frontiers in Veterinary Science 5; 2018. 10.3389/fvets.2018.00069 PMC5906542 29707545

[7] Edwards FW. Mosquitoes of the Ethiopian Region III. – Culicine adults and pupae. Printed by order of the Trustees, British Museum (Natural History), London; 1941.

[8] Elliott R, Weber F (2009). Bunyaviruses and the Type I interferon system. Viruses.

[9] Gerrard SR, Li L, Barrett AD, Nichol ST (2004). Ngari virus is a Bunyamwera virus reassortant that can be associated with large outbreaks of hemorrhagic fever in Africa. J Virol.

[10] Gillies MT, Coetzee M. A supplement to the Anophelinae of Africa south of the Sahara (Afrotropical Region). Publications of the South African Institute for Medical Research 55; 1987.

[11] Groseth A, Weisend C, Ebihara H (2012). Complete genome sequencing of mosquito and human isolates of Ngari Virus. J Virol.

[12] Groseth A, Mampilli V, Weisend C, Dahlstrom E, Porcella SF, Russell BJ, Tesh RB, Ebihara H (2014). Molecular Characterization of Human Pathogenic Bunyaviruses of the Nyando and Bwamba/Pongola Virus Groups Leads to the Genetic Identification of Mojuí dos Campos and Kaeng Khoi Virus. PLOS Neglect Trop Diseases.

[13] Guu TS, Zheng W, Tao YJ (2012). Bunyavirus: structure and replication. Adv Exp Med Biol.

[14] Harbach RE. The mosquitoes of the subgenus Culex in southwestern Asia and Egypt (Diptera: Culicidae). Contributions of the American Entomological Institute 24; 1988.

[15] Hall TA. BioEdit: a user-friendly biological sequence alignment editor and analysis program for Windows 95/98/NT. Nucleic acids symposium series. [London]: Information Retrieval Ltd., c1979-c2000., pp. 95–98; 1999.

[16] Huhtamo E, Lambert AJ, Costantino S, Servino L, Krizmancic L, Boldorini R, Allegrini S, Grasso I, Korhonen EM, Vapalahti O, Lanciotti RS, Ravanini P (2013). Isolation and full genomic characterization of Batai virus from mosquitoes, Italy 2009. J Gen Virol.

[17] Jöst H, Bialonski A, Schmetz C, Günther S, Becker N, Schmidt-Chanasit J (2011). Isolation and phylogenetic analysis of Batai virus, Germany. Am J Trop Med Hyg.

[18] Kokernot RH, Heymann CS, Muspratt J, Wolstenholme B (1957). Studies on arthropod-borne viruses of Tongaland. V. Isolation of Bunyamwera and Rift Valley Fever viruses from mosquitoes. S Afr J Med Sci.

[19] Kokernot RH, Smithburn KC, De Meillon B, Paterson HE (1958). Isolation of Bunyamwera virus from a naturally infected human being and further isolations from Aedes (Banksinella) circumluteolus theo. Am J Trop Med Hyg.

[20] Konongoi SL, Nyunja A, Ofula V, Owaka S, Koka H, Koskei E, Eyase F, Langat D, Mancuso J, Lutomiah J, Sang R (2018). Human and entomologic investigations of chikungunya outbreak in Mandera, Northeastern Kenya, 2016. PLoS ONE.

[21] Kumar S, Stecher G, Tamura K (2016). MEGA7: molecular evolutionary genetics analysis version 7.0 for bigger datasets. Mol Biol Evol.

[22] Kuno G, Mitchell CJ, Chang GJ, Smith GC (1996). Detecting bunyaviruses of the Bunyamwera and California serogroups by a PCR technique. J Clin Microbiol.

[23] Lutomiah J, Barrera R, Makio A, Mutisya J, Koka H, Owaka S, Koskei E, Nyunja A, Eyase F, Coldren R, Sang R (2016). Dengue outbreak in Mombasa City, Kenya, 2013–2014: Entomologic investigations. PLoS Neglect Trop Diseases.

[24] Lwande OW, Lutomiah J, Obanda V, Gakuya F, Mutisya J, Mulwa F, Michuki G, Chepkorir E, Fischer A, Venter M, Sang R (2013). Isolation of tick and mosquito-borne arboviruses from ticks sampled from livestock and wild animal hosts in Ijara District, Kenya. Vector Borne Zoonotic Dis.

[25] Nashed NW, Olson JG, el-Tigani, A.,  (1993). Isolation of Batai virus (Bunyaviridae:Bunyavirus) from the blood of suspected malaria patients in Sudan. Am J Trop Med Hyg.

[26] ngs_mapper, V.W., Genome Mapping pipeline-Github.

[27] Ochieng C, Lutomiah J, Makio A, Koka H, Chepkorir E, Yalwala S, Mutisya J, Musila L, Khamadi S, Richardson J, Bast J, Schnabel D, Wurapa E, Sang R (2013). Mosquito-borne arbovirus surveillance at selected sites in diverse ecological zones of Kenya; 2007–2012. Virol J.

[28] Odhiambo C, Venter M, Swanepoel R, Sang R (2015). Orthobunyavirus antibodies among humans in selected parts of the Rift Valley and Northeastern Kenya. Vector Borne Zoonotic Diseases (Larchmont, N. Y.).

[29] PanAmerican OH. Dengue and dengue hemorrhagic fever in the Americas: guidelines for prevention and control. Pan Amer Health Org; 1994.

[30] RCoreTeam. R: A language and environment for statistical computing. R Foundation for Statistical Computing. Vienna, Austria; 2019.

[31] Sanchez L, Vanlerberghe V, Alfonso L, Marquetti, M.d.C., Guzman, M.G., Bisset, J., van der Stuyft, P.,  (2006). Aedes aegypti larval indices and risk for dengue epidemics. Emerg Infect Dis.

[32] Sang R, Kioko E, Lutomiah J, Warigia M, Ochieng C, O'Guinn M, Lee JS, Koka H, Godsey M, Hoel D, Hanafi H, Miller B, Schnabel D, Breiman RF, Richardson J (2010). Rift Valley fever virus epidemic in Kenya, 2006/2007: The entomologic investigations. Am J Trop Med Hyg.

[33] Scheuch DE, Schäfer M, Eiden M, Heym EC, Ziegler U, Walther D, Schmidt-Chanasit J, Keller M, Groschup MH, Kampen H (2018). Detection of Usutu, Sindbis, and Batai Viruses in Mosquitoes (Diptera: Culicidae) Collected in Germany, 2011–2016. Viruses.

[34] SPACES, 2012. Sustainable Poverty Alleviation from Coastal Ecosystem Services.

[35] Smithburn KC, Haddow AJ, Mahaffy AF (1946). A neurotropic virus isolated from Aedes mosquitoes caught in the Semliki forest. Am J Trop Med Hyg.

[36] Suwanbamrung C (2018). Developing the active larval indices surveillance system for dengue solution in low and high dengue risk primary care units, Southern Thailand. J Health Res.

[37] Udayanga L, Gunathilaka N, Iqbal MCM, Najim MMM, Pahalagedara K, Abeyewickreme W (2018). Empirical optimization of risk thresholds for dengue: an approach towards entomological management of Aedes mosquitoes based on larval indices in the Kandy District of Sri Lanka. Parasit Vectors.

[38] Wang J-N, Hou J, Zhong J-Y, Cao G-P, Yu Z-Y, Wu Y-Y, Li T-Q, Liu Q-M, Williams MC, Woodall JP, Corbet PS (1965). Nyando Virus: A Hitherto Undescribed Virus Isolated From Anopheles Funestus Giles Collected In Kenya. Archiv fur die gesamte Virusforschung.

[39] WorldBank. An environmental and social impact assessment ESIA report in Kwale Town Water Supply Expansion and Rehabiltation Project; 2019.

[40] Yanase T, Kato T, Yamakawa M, Takayoshi K, Nakamura K, Kokuba T, Tsuda T (2006). Genetic characterization of Batai virus indicates a genomic reassortment between orthobunyaviruses in nature. Adv Virol.

[41] Yandoko EN, Gribaldo S, Finance C, Le Faou A, Rihn BH (2007). Molecular characterization of African orthobunyaviruses. J Gen Virol.

[42] Zeller HG, Diallo M, Angel G, Traoré-Lamizana M, Thonnon J, Digoutte JP, Fontenille D (1996). Ngari virus (Bunyaviridae: Bunyavirus). First isolation from humans in Senegal, new mosquito vectors, its epidemiology. Bull Soc Pathol Exot.

[43] Zhang L, Zhang Q, Wang J, An N, Cao Y, Fu G, Hu X, Huang Y, Su J (2017). Characterization of Batai virus isolated from a domestic Muscovy duck (*Cairina moschate*). Virus Genes.

